# Occupational Exposure to Fine Particulate Matter (PM_4_ and PM_2.5_) during Hand-Made Cookware Operation: Personal, Indoor and Outdoor Levels

**DOI:** 10.3390/ijerph17207522

**Published:** 2020-10-16

**Authors:** Busisiwe Shezi, Angela Mathee, Nokulunga Cele, Sipho Ndabandaba, Renee A. Street

**Affiliations:** 1 Environment and Health Research Unit, South African Medical Research Council, Johannesburg 2094, South Africa; Angie.Mathee@mrc.ac.za; 2 Department of Environmental Health, Faculty of Health Sciences, University of Johannesburg, Johannesburg 2094, South Africa; 3 Department of Environmental Health, Nelson Mandela University, Port Elizabeth 6019, South Africa; 4 Environment and Health Research Unit, South African Medical Research Council, Durban 4001, South Africa; nokulungacele6@gmail.com (N.C.); ndabandabasp@gmail.com (S.N.); Renee.Street@mrc.ac.za (R.A.S.); 5 University of KwaZulu-Natal, Discipline of Occupational and Environmental Health, School of Nursing and Public Health, Durban 4001, South Africa

**Keywords:** hand-made cookware operation, aluminum, personal exposure, microenvironment assessment, cottage industry, informal sector, fine particles

## Abstract

(1) Exposure of informal artisanal cookware makers to fine particles has not yet been characterized. The aim of this study was to characterize occupational exposure to fine particulate matter (PM_4_ and PM_2.5_) levels and fine particulate matter (PM_2.5_) elemental components; (2) Artisanal cookware makers were recruited from five cookware making sites. Exposure to fine particulate matter was measured for 17 male participants. SidePak personal aerosol monitors (AM520) were used to measure personal exposure to PM_4_, while a DustTrak monitor and an E-sampler were used to assess indoor and outdoor PM_2.5_ levels, respectively. A questionnaire was administered to capture information on demographic characteristics. The chemical characterization of indoor and outdoor PM_2.5_ filter mass was conducted using Wavelength Dispersive X-ray Fluorescence. Time series record of 15-min averages for indoor and outdoor PM_2.5_ levels were assessed; (3) The median (range) was 124 µg/m^3^ (23−100,000), 64 µg/m^3^ (1−6097) and 12 µg/m^3^ (4−1178), respectively, for personal PM_4_, indoor and outdoor PM_2.5_. The highest levels for many of the elemental components of PM_2.5_ were found in the outdoor PM_2.5_ filter mass and (4). The information generated during this study may assist in extending occupational health and safety strategies to artisanal cookware makers and developing targeted prevention initiatives.

## 1. Introduction

Epidemiological studies have consistently found significant positive associations between airborne particulate matter (PM) and a variety of adverse health outcomes, including respiratory and cardiovascular illnesses [1,2,3] and adverse pregnancy outcomes [4,5,6,7]. According to the World Health Organization, PM affects more people than any other pollutant [8]. In addition, long term health effects of exposure to PM outweigh short term health effects with regards to public health significance [9]. PM is defined by the United States Environmental Protection Agency as “a mixture of solid and liquid droplets suspended in the air such as organic chemicals, soot, metals, soils or dust particles and biological material” [10]. PM may contain a mixture of several pollutants distributed at various sizes including PM_0.1_ (aerodynamic diameter less than 0.1 micrometers), PM_2.5_ (aerodynamic diameter less than 2.5 micrometers) or PM_4_ (aerodynamic diameter less than 4 micrometers) [11]. 

In occupational settings, exposure to PM may arise from, for example, agricultural activities [12], boiler making [13], metal mining [14,15,16,17], and informal, home-based industrial operations [18]. Informal, home-based industries include spray painting [19], welding [20,21,22], electrical appliance repairs [23] and hand-made cookware operations [24,25]. Recently, concerns have been raised about the harmful contents of locally available hand-made cookware [26,27]. For example, studies conducted in Cameroon [26] and South Africa [27] have reported the use of toxic metals during the hand-made cookware operation, including lead and cadmium. Similar findings have been reported in West African countries, i.e., Nigeria [24,25]. However, there is a dearth of information about exposure to toxic substances among cookware makers during the hand-made cookware making process. Artisanal cookware makers use scrap metal including used car and motorbike engine parts, waste aluminum and computer components to make cookware [24,25]. It is well known that PM and its elemental components are virtually always present in particle-generating processes, especially combustion processes [28]. For example, during hand-made cookware operations, PM and elemental components are produced through the combustion process to cast liquid aluminum into cookware [24,26]. In addition, cookware makers are exposed to the latter during the sand mold production, solidification monitoring and removing and trimming the casting [25,29]. 

Occupational health and safety is essential for the well-being of both formal (regulated, registered and protected by the labor legislation) and informal (not recognized, registered, regulated and/or protected under labor legislation and social protection) workers [30]. However, the use of administrative controls (i.e., job rotation), engineering controls and personal protective equipment is not always practiced in informal settings [31]. Further, little attention has been given to the effects of occupational exposures, which differ from general environmental exposures in both particle type (e.g., composition), as well as exposure frequency (e.g., environmental exposures are relatively constant while occupational exposures are more variable), duration, intensity or levels (e.g., occupational exposures are generally higher than environmental exposures). These differences in exposure composition, duration, frequency and population may have implications on how occupational exposures impact health. 

There are limited occupational health studies where personal, indoor and outdoor PM have been characterized and compared, especially among informal workers, and none to our knowledge in artisanal cookware makers. In order to get a better understanding of the health risks posed by PM among artisanal cookware makers, we measured occupational exposure to fine particulate matter (PM_4_ and PM_2.5_) during hand-made cookware operation and assessed the chemical characterization of indoor and outdoor PM_2.5_ filter mass. The relationship between personal, indoor and outdoor PM levels was also examined.

## 2. Materials and Methods 

### 2.1. Study Design, Setting and Population

This study was undertaken during the months of June and July 2019. The target population was artisanal cookware makers situated in the province of Limpopo (Giyani) and Kwa-Zulu Natal (Durban), South Africa. Giyani is a city in the north-eastern region of the Limpopo province of South Africa and Durban is situated along the east coast of the country. Signed informed consent was obtained from all participants. The study was approved by the University of KwaZulu-Natal Biomedical Research Ethics Committee (reference number: BE410/18).

### 2.2. Exposure Assessment 

**Figure 1:**
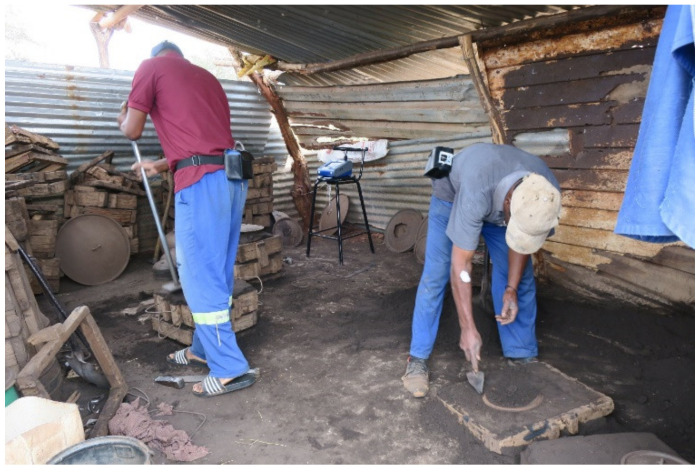
One of the hand-made cookware operation sites with artisans preparing sand mold.

Seventeen artisanal cookware makers were assessed for personal exposure to PM_4_. Five hand-made cookware operation sites were monitored for PM_2.5_ (Figure 1 shows one of the hand-made cookware operation sites); however, outdoor and/or indoor measurements were incomplete in one site (there was no electricity to connect the E-sampler and unwillingness to participate for the entire duration of indoor sampling period led to incomplete measurements). A questionnaire was administered to capture demographic characteristics such as age, marital status, tobacco smoking and education. The questionnaires were captured face-to-face, by field workers. Study data were collected and managed using REDCap electronic data capture tools. 

### 2.3. Personal Air Monitoring 

A portable, real time photometric aerosol monitor (SidePak^TM^ TSI model AM520, TSI Inc., Shoreview, MN, USA) was used to measure PM_4_ levels among 17 adult males making artisanal cookware. The monitor was carried by the cookware makers for a period of 3 h at a flow rate of 1.7 L/min. The breathing zone of participants was assessed using a 10-mm Nylon Dorr-Oliver Cyclone inlet (TSI Inc., Shoreview, MN, USA) which differentiates between the respirable fraction and other portions of PM. The cyclone was attached to the worker’s clothing near his head. The logging interval was set at 1 min. The SidePak monitors were calibrated prior to the data collection phase using the guidelines set by the manufacturer. 

### 2.4. Indoor Air Monitoring 

A DustTrak (TSI Inc., Shoreview, MN, USA) photometric light scattering monitor which had been calibrated one year before the sampling period was used to measure indoor PM_2.5_. Both real time and mass sampling (using mixed cellulose ester membrane filters, 37 mm (SKC Ltd, Dorset, UK)) were used for sampling indoor PM_2.5_ (for a period of 8 h). The sampler flow rate was set at 3 L/min using a 1 min logging interval. A suitable location (at least 0.5 m from the wall and away from the door or gap between the wall and the roof) was identified for placing the DustTrak monitor.

### 2.5. Outdoor Air Monitoring

An E-Sampler (Met One Instruments, Grants Pass Oregon) was used to assess outdoor PM_2.5_ for a period of 8 h. The E-sampler is designed to measure the amount of scattered light and uses a conversion factor (indicated by K) to convert light scattering to mass. Therefore, both real time (1-min logging interval) and mass sampling were conducted. Mixed cellulose ester membrane filters, 47 mm (SKC Ltd, Dorset, UK) were used for sampling outdoor PM_2.5_. The sampler flow rate was set at 2 L/min. The E-sampler calculated the K constant during operation and self-adjusted itself. The E-sampler has internal temperature and relative humidity sensors to autocorrect for high atmospheric water content, which can influence the measurements.

### 2.6. Gravimetric Analysis

Gravimetric analysis was used to weigh filters prior to sampling using an XP26 DeltaRange Microbalance (Mettler-Toledo AG, Greifensee, Switzerland) accurate to 1 microgram. The 37- and 47-mm filters were conditioned for 24 h in the laboratory with controlled indoor climate of 22 °C ± 2 °C and relative humidity (not captured). After sample collection, the filters were returned to the petri dishes, conditioned, re-weighed and stored at 4 °C until they could be chemically analyzed. The PM_2.5_ levels were calculated using the following equation:

PM_2.5_ µg/m^3^= ([(Wf-Wi)-blank filter mass] × 10^−6^)/Vt (1)

where Wf is the filter mass after sampling and Wi is the filter mass before sampling in grams (g). The blank filter is the change in mass of the blank filter calculated by weighing the blanks before and after sampling and is expressed in grams (g). Vt is the total volume of sampled air during the 8-h period. Total volume was calculated using the sampled flow rate, ambient temperature and pressure during sampling. Each site had around (915 L (0.9 m^3^): Esampler), and (1395 L (1.4 m^3^): DustTrak) sampled air over the 8-h period. The resulting concentration was calculated in µg/m^3^.

### 2.7. PM_2.5_ Chemical Characterization

For additional information on elemental composition of the indoor and outdoor PM_2.5_ filter mass, the filters were subsequently analyzed for 49 elements at the University of North West, South Africa, using wavelength-dispersive x-ray fluorescence (WD-XRF) technique. The following elements were analyzed Ag, Al, As, Au, Ba, Bi, Br, Ca, Ce, Cd, CI, Co, Cr, Cs, Cu, Fe, Ga, Ge, Hg, I, In, K, Li, Mg, Mn, Mo, Na, Nb, Ni, P, Pb, Pd, Pt, Rb, S, Sb, Sc, Se, Si, Sn, Sr, Te, Ti, Tl, V, W, Y, Zn and Zr.

### 2.8. Quality Assurance and Control

The SidePak and DustTrak monitors were factory calibrated according to the ISO 12103−1, A1 Arizona test dust. Prior to data collection, the latter instruments were zero calibrated by attaching the zero-filter, as recommended by the manufacturer. The batteries were charged on a daily basis to maintain battery capability during sampling and the impactor plates were checked and cleaned on a daily basis. The output for personal, indoor and outdoor measurements was given in milligram per cubic meter (mg/m^3^) but converted to microgram per cubic meter (µg/m^3^) prior to data analysis. Data were downloaded from the instruments after every sampling period using the TSI Trackpro (DustTrak and SidePak) and Comet (E-sampler). A standardized log sheet was used to record sampling start and stop times. Laboratory and field blank filters (indoor (*n* = 4), outdoor (*n* = 4)) were used to adjust the weight difference observed due to changes in indoor climate of the weighing room and field handling of samples, respectively. The field blanks (indoor (*n* = 2), outdoor (*n* = 2) were taken to the hand-made cookware operation sites and were handled the same way as the other filters (i.e., the field blank filters were loaded into the sampler for five minutes (no air was drawn through the blank filters). The limit of detection (LOD) was estimated using the method by Vaughan et al., [32] using the mean of standard deviation of the mass change of blank filters multiplied by three. Standardized field sampling flow rates for the E-sampler and DustTrak were used. The LOD divided by the square root of 2 was used in all calculations [33]. All indoor and outdoor values were above the limit of detection. Invalid PM levels were identified by inspection of plots; a small number of cases when the monitor appeared to be performing inaccurately were noted. Zero and negative values were excluded from the dataset. The indoor and outdoor PM_2.5_ photometric measurements were adjusted by using the calibration factor to approximate the actual PM_2.5_ mass levels.

## 3. Statistical Analysis

All data were cleaned and checked for quality on Microsoft Excel and exported to Stata IC version 14 (StataCorp, College Station, TX, USA) for further analysis. Statistical analysis was restricted to those observations that had concurrent levels of personal, indoor and outdoor air pollution measurements available. Descriptive statistics, scatterplots, and histograms were used to characterize distributions of PM levels. Quartiles, means (standard deviations) and medians (range) for PM were used. Real time data were collected every 1 min, but 15-min averages were used for analysis.

The 17 workers who were assessed for 3-h personal sampling were categorized into three groups: (i) 1st PM_4_ sampling sessions; workers assessed for personal sampling in the morning, (ii) 2nd PM_4_ sampling sessions; workers assessed for personal sampling just before midday, and (iii) 3rd PM_4_ sampling sessions; workers assessed for personal sampling in the afternoon. Concurrent 3-h measurements of indoor and outdoor PM_2.5_ measurements were matched with the 3-h PM_4_ personal measurements. Four workers were excluded from this analysis because of incomplete/no PM_2.5_ measurements to match with PM_4_ levels. The relationship between 3-h personal, indoor and outdoor measurements was assessed using Spearman’s rank order correlation.

The relationship between indoor and outdoor PM_2.5_ levels was examined using time series indoor-outdoor plots and by calculating Spearman’s rank order correlation using the 15-min averages of PM.

## 4. Results

### 4.1. Demographic and Site Characteristics

All 17 cookware makers were male. The mean (SD) for participant age was 34 years (11). The majority (59%) were single and had attended or completed high school (65%). Wood was used in three of the five sites and in two sites artisans used coal for melting aluminum. All sites had a gap between the wall and the roof and were built with corrugated metal sheeting.

Two sites were situated in low socio-economic urban areas and three in rural areas. Four of the five sites were on residential plots. Descriptions of the sites sampled and the number of workers who participated in PM sampling are summarized in Table 1.

### 4.2. Real Time Personal, Indoor and Outdoor PM Measurements

PM levels in our study were high (Table 2). The real time personal PM_4_, indoor and outdoor PM_2.5_ levels were negatively skewed. The levels ranged from 23 to 100,000 µg/m^3^; 1 to 6097 µg/m^3^ and 1 to 1178 µg/m^3^, respectively, for personal PM_4_, indoor and outdoor PM_2.5_ levels. When comparing personal PM_4_ and indoor and outdoor PM_2.5_ levels, personal exposure levels were found to be the highest, mean (SD) was 492 µg/m^3^ (3546), with a median of 124 µg/m^3^, followed by workplace indoors, mean (SD), 98 µg/m^3^ (262), with a median of 64 µg/m^3^. The mean (SD) and median for workplace outdoors was 20 µg/m^3^ (45) and the median was 13 µg/m^3^ (Table 2).

### 4.3. Indoor and Outdoor PM_2.5_ Concentration Measurements

The mass measurements for indoor and outdoor PM_2.5_ levels ranged from 6 to 371 µg/m^3^ and 8.8 to 51 µg/m^3^, respectively. The mean (SD) was 105 µg/m^3^ (137), and 19 µg/m^3^ (16); and the median was 61 µg/m^3^ and 14 µg/m^3^, respectively, for indoor and outdoor measurements.

### 4.4. Meteorological Data

Outdoor temperature was mainly in the range of 16 to 38 °C, with a mean (SD) of 26 °C (5). Relative humidity ranged from 6% to 55% with a mean (SD) of 29% (11) (Table 2).

### 4.5. Personal, Indoor and Outdoor Relationships (3-h Measurements)

Personal–indoor and personal–outdoor Spearman correlations (*r*) of 3-h concurrent measurements were poor for both the 1st and 2nd PM sampling session. Personal–indoor correlations and personal–outdoor correlations were (*r* =0.05, *p* > 0.05), (*r* =−0.18; *p* > 0.05), and (*r* = −0.08, *p* > 0.05), (*r* = −0.37, *p* > 0.05), respectively, for workers sampled in the 1st and 2nd PM sampling session.

### 4.6. Indoor and Outdoor Relationships (8-h Measurements)

Correlations between indoor–outdoor PM_2.5_ samples, indoor PM_2.5_–outdoor meteorological factors and outdoor PM_2.5_–outdoor meteorological factors are shown in Table 3. Indoor–outdoor PM_2.5_ correlations were moderate for site 2 (*r* = 0.67, *p* < 0.0001). For site 1, 2 and 3 the indoor–outdoor PM_2.5_ correlations were (*r* = −0.32, *p* < 0.05), (*r* = 0.28, *p* > 0.05) and (*r* = 0.43, *p* < 0.05), respectively.

For all sites, outdoor PM_2.5_ levels were negatively correlated with outdoor temperature. This correlation ranged from weak (*r* = −0.15) to high (*r* = −0.85). Correlation between indoor PM_2.5_ levels and outdoor temperature was also negatively correlated in three sites. The correlation was moderate (*r* = −0.39, *p* > 0.05) to high (*r* = −0.73, *p* < 0.05). All sites had a positive correlation between microenvironmental levels and humidity except an insignificant weak negative correlation between indoor PM_2.5_ levels and humidity observed in site 1 (*r* = −0.07, *p* > 0.05).

### 4.7. Time Series Record of 15-min Average Indoor and Outdoor PM_2.5_ Real Time Measurements

**Figure 2:**
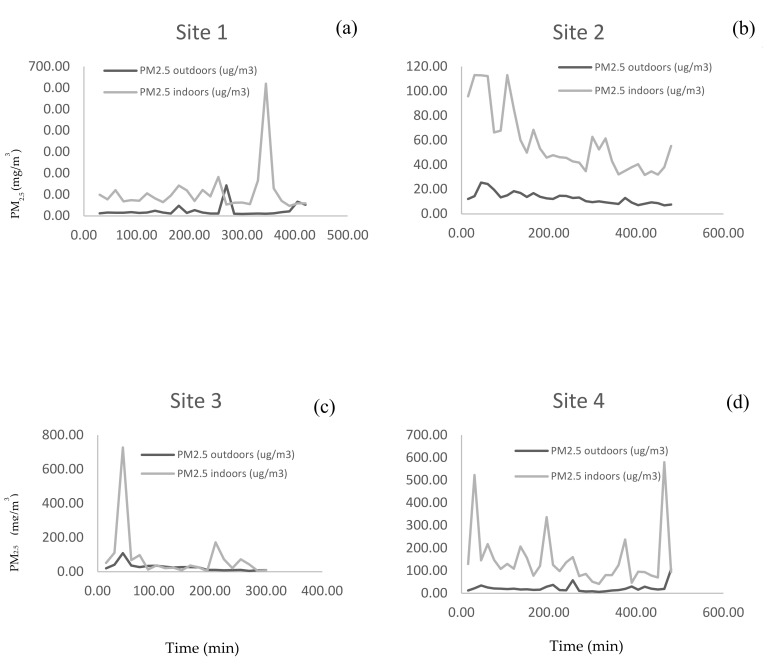
A comparison of time series record of 15-min average indoor and outdoor PM_2.5_ real-time measurements (**a**) Site 1: five cookware makers occupied this site, and it was situated in rural areas and wood was used in furnaces; (**b**) Site 2: two cookware makers occupied this site, and it was situated in rural areas and wood was used for furnace; (**c**) Site 3: thirteen workers occupied this site, and it was situated in the urban area and coal was used for furnace; (**d**) Site 4: 6 workers occupied this site, and it was situated in urban areas and wood was used for furnace.

The relationship between indoor and outdoor PM_2.5_ real time levels is shown in Figure 2. In all sites indoor PM_2.5_ levels remained higher than outdoor levels with little evidence of strong outdoor sources. There were frequent large indoor peaks reflecting significant indoor sources. Across all sites average indoor 15-min levels were variable than the outdoor levels.

### 4.8. PM_2.5_ Elemental Components

Out of 49 indoor and outdoor analyzed elements, thirteen (Ag, Au, Bi, Co, Ga, Ge, Hg, Li, Mo, Nb, Se, Sn, and Tl) had zero values and were excluded in the results. The results for 38 elements are shown in Table 4. The highest levels for many of the elemental components of PM_2.5_ were found in the outdoor filter mass (Table 4). The elemental components for both indoor and outdoor PM_2.5_ were much lower than the occupational exposure limit recommended by the South African regulations for Hazardous Chemical Substances and Lead regulations under the Occupational Health and Safety (OHS) Act (Act 85 of 1993).

Some of the PM elements are not reported in the regulation for Hazardous Chemical Substances under the OHS Act (Act 85 of 1993).

## 5. Discussion

In this study, we described personal (PM_4_), indoor and outdoor (PM_2.5_) levels and PM_2.5_ elemental components among artisanal cookware makers. The relationship between personal, indoor and outdoor measurements was assessed. Our data are consistent with the general pattern observed in most PM monitoring studies [34,35,36,37]: outdoor levels are lower than indoor levels, and both indoor and outdoor levels are lower than personal levels. Elemental components in outdoor PM_2.5_ filter mass are higher than indoors. These findings strengthen our understanding of the worker–exposure relationship during hand-made cookware operations, especially with the substantial differences observed between personal, indoor and outdoor measurements.

Particulate matter is associated with respiratory effects and chronic obstructive pulmonary diseases [2,3,38] and has been reported to result in increased hospital admissions, outpatient visits and emergency admissions [39,40]. Other studies have reported an inverse association between particulate matter and adverse pregnancy outcomes such as birth defects, low birth weight and preterm delivery [41,42]. Overall, the levels of fine particles found in our study were high. The air quality guidelines for PM_2.5_ suggested by the WHO expert group are 10 µg/m^3^ as an annual mean and 25 µg/m^3^ as a 24-h mean [43]. In this study we obtained mean levels of 98 and 20 µg/m^3^ for indoor and outdoor measurements (8-h mean) of PM_2.5_, respectively. We compared our personal measurements with the existing occupational exposure limits in South Africa. According to the regulations for Hazardous Chemical Substances, under the OHS Act (Act 85 of 1993), the personal exposure limit for respiratory dust is 5000 µg/m^3^ (average 8-h respiratory dust). In our study, the 3-h time weighed average concentrations ranged from 42 to 300 µg/m^3^; therefore, they were much lower than the occupational exposure limit. However, in site 5 we observed very high concentrations of PM_4_ (ranging between 5010 and 100,000 µg/m^3^), this might have been because coal was used for furnace in this site.

Exposure of artisanal cookware makers during cookware making is undocumented in the literature. Some studies conducted previously have shown that microenvironment assessment does not always reflect personal exposure [34,35,36]. Similarly, concurrent samples undertaken in this study revealed that the averaged personal exposure levels (3-h mean) at breathing zones of workers are much greater (490 µg/m^3^) than those of the microenvironment levels. Though the comparisons were based on two different fractions of PM (PM_4_ and PM_2.5_) and despite the undocumented exposures among artisanal cookware makers, other studies have reported similar findings [34,36]. The high personal PM_4_ levels observed in this study might be related to frequent mobility of the workers within their working areas. Furthermore, various activities of the workers such as design and production of the mold for the cookware, pouring of melted aluminum into the mold cavity, solidification monitoring, removing and trimming the casting [25,29] could significantly increase the PM levels.

In all the sites, indoor PM_2.5_ levels remained higher than outdoor levels with little evidence of strong outdoor sources. However, we also observed considerable differences between the sites, for example, frequent large indoor peaks were observed in some of the sites. This could be explained by the differences in activity levels, the number of workers per site and the size of the working site (which ranged from 35 to 127 m^3^). In addition, natural ventilation in all the sites was in the form of a gap between the wall and the roof, allowing particles from outside to readily penetrate into the workplace. Therefore, another possible explanation for higher indoor levels may be the infiltration of outdoor pollutants indoors, which may be influenced by wind speed and wind direction [44].

In our study, the cookware making sites were situated on residential plots, and therefore exposed groups may include the entire family, neighbors and the cookware makers. Therefore, the measurements of indoor and outdoor PM_2.5_ levels provided an indication of how the workers are exposed to PM_2.5_ and the potential impacts this might have to the families living in these sites.

A weak correlation was observed between personal–indoor and personal–outdoor levels. This could be explained by the variations in time spent indoors and outdoors by the workers, differences in ventilation and distance between the cookware making area and furnace across the sites. Similar findings have been observed from other studies comparing indoor, outdoor and personal measurements in occupational settings [45,46].

Epidemiological studies have reported that particle-bound metals are associated with mitochondrial damage [47] and induction of oxidative stress [48], which results in an increase in cardiovascular mortality and morbidity [49]. These elements may originate from various industrial or urban sources. In our study area, the mean outdoor metal levels were higher than indoors. This may be as a result of the outdoor furnace, where various types of metal containing products are used, i.e., e-waste (from computer parts), vehicle and motor bike engine parts. Even so, when comparing our study findings with the occupational exposure limit reported under the Hazardous Chemical Substances Regulation (1995) and Lead Regulations (2001) of the OHS Act (Act 85 of 1993), our results were much lower.

One of the study limitations was that a convenience sample of artisanal cookware makers was drawn, which limits our ability to generalize our findings. In our study, different sampling equipment was used for personal, indoor and outdoor PM levels. A good agreement between the real-time measurements and the particle mass measurements was shown by the overall distribution of PM_2.5_ mass levels, which was comparable to the distribution of real-time PM_2.5_ levels. Unlike levels of PM_2.5_ and PM_10_, concurrent levels of PM_4_ and PM_2.5_ have rarely been investigated in workplaces. By taking parallel measurements of PM_4_ and PM_2.5_ we were able to compare levels of the two size fractions; however, PM_2.5_ fraction is a fraction of PM_4_, so the quantities cannot be compared equivalently. Because of the small sample size, we could not assess the association between PM and exposure variables such as smoking. However, in our study the number of participants who were active smokers was limited. Therefore, the impact of tobacco smoke on personal, indoor and outdoor levels is likely to be limited. Light scattering photometers have been reported to result in overestimation of the PM concentrations when compared to a gravimetric method. In our study, we used light scattering photometers and gravimetric analysis. To obtain accurate gravimetric analysis of PM, temperature stability, extreme care exercise in sample handling and moisture control is required [50]. However, relative humidity was not captured by the laboratory for our study. Therefore, it is not clear whether the relative humidity was within the recommended limit or not during our study. The performance of PM monitors varies because of the differences in technology, therefore, using different monitors may limit the comparison of the same fraction of PM. In our study we used different instruments for measuring personal, indoor and outdoor PM.

## 6. Conclusions

This to our knowledge is the first study to assess PM during hand-made cookware operations. The levels measured at the breathing zone of the workers were higher than the levels from indoors and outdoors. There were frequent large indoor peaks reflecting significant indoor sources. The chemical characterization of indoor and outdoor PM filter mass provided metal levels and the mean outdoor elemental levels were relatively higher than mean indoor elemental levels. According to our results and those of many others, levels measured at microenvironment level are a poor predictor of personal (cross-sectional short-term) workday exposure (from all sources). Therefore, identification of occupational exposure during hand-made cookware operation allows for more inclusive occupational health and safety strategies.

## Figures and Tables

**Table 1:** Description of the sites and workers sampled.

Site No.	Total No. of Workers	1st PM_4_ Sampling Session	2nd PM_4_ Sampling Session	3rd PM_4_ Sampling Session	Indoor Sampling	Outdoor Sampling	Volume of the Site (m^3^)
**1**	5	2	1	-	Yes	Yes	42.5
**2**	2	2	-	-	Yes	Yes	34.9
**3**	5	2	-	-	Yes	Yes	77.8
**4**	13	2	2	2	Yes	Yes	127.0
* 5	6	2	2	-	Yes (incomplete)	No	71.7

* There was no electricity to connect the E-sampler and unwillingness to participate for the entire indoor 8-h sampling period led to incomplete measurements.

**Table 2:** Particulate matter (PM) measurements and meteorological data.

Levels	Personal PM_4_ (µg/m^3^)	Indoor PM_2.5_ (µg/m^3^)	Outdoor PM_2.5_ (µg/m^3^)	Outdoor Temperature (°C)	Outdoor Humidity (%)
Min	23	1	3.6	16	6
10th percentile	74	31	7	18	15
25th percentile	90	44	9	23	22
50th percentile	124	64	13	26	27
75th percentile	182	99	19	28	36
Max	100 000	6097	1178	38	55
Mean (SD)	492 (3546)	98 (262)	20 (45)	26 (5)	29 (11)

Abbreviations: SD (standard deviation); µg/m^3^ (micrograms per cubic meter).

**Table 3:** The Spearman’s rank order correlation for 15-min averages of indoor and outdoor PM_2.5_ levels, outdoor temperature and outdoor humidity.

Site No.	Indoor/Outdoor PM_2.5_ Levels	Indoor PM_2.5_/Temperature	Outdoor PM_2.5_/Temperature	Indoor PM_2.5_/Humidity	Outdoor PM_2.5_/Humidity
1	−0.32	0.08	−0.15	−0.07	0.37
2	**0.67**	**−0.73**	**−0.73**	**0.80**	**0.84**
3	0.28	−0.39	**−0.85**	0.33	**0.88**
4	**0.43**	**−0.53**	**−0.48**	**0.51**	**0.50**

Bolded values indicate significance at 95% confidence interval (95% CI).

**Table 4:** Metal levels of indoor and outdoor PM_2.5_ filter mass.

Sample Name	Indoor (µg/m^3^)	Outdoor (µg/m^3^)	*8-h TWA OEL-RL (µg/m^3^)
Al	0.2	1.9	10500
As	1.0	2.5	100
Ba	0.1	0.2	500
Br	0.0	0.2	100
Ca	1.4	1.4	-
Ce	0.4	0.9	-
Cd	0.1	0.0	50
CI	1.9	3.2	-
Cr	0.4	0.6	50
Cs	0.3	0.5	
Cu	4.0	7.1	1000
Fe	0.9	1.9	-
I	0.2	0.4	-
In	0.9	0.7	-
K	0.3	2.9	-
Mg	0.1	1.3	5100
Mn	0.1	0.6	5000
Na	7.7	7.4	-
Ni	0.4	0.8	50
P	0.0	0.1	100
Pb	2.8	6.6	150
Pd	71.8	162.2	-
Pt	12.2	28.7	5000
Rb	0.2	0.2	-
S	0.2	0.1	-
Sb	1.9	4.8	-
Sc	0.1	0.2	-
Si	0.8	7.4	100
Sr	0.2	0.0	-
Te	8.2	18.3	100
Ti	0.0	0.1	-
V	0.0	0.1	-
W	0.0	0.1	5000
Y	0.0	0.2	1000
Zn	5.3	9.7	
Zr	0.2	0.1	5000

* TWA OEL-RL: Time Weighted Average Occupational Exposure Limit-Recommended Limit.
